# Herpes simplex virus 1 and 2 educational assessment of young adults in rural southwest Virginia

**DOI:** 10.1371/journal.pone.0179969

**Published:** 2017-06-27

**Authors:** Shantal S. Hover, Andrea S. Bertke

**Affiliations:** Department of Population Health Sciences, Virginia Polytechnic Institute and State University, Blacksburg, VA, United States of America; Cincinnati Children's Hospital Medical Center, UNITED STATES

## Abstract

**Purpose:**

Herpes simplex virus 2 (HSV-2) causes genital herpes, one of the most common sexually transmitted infections (STIs) in the U.S. HSV-1, commonly associated with cold sores, is increasing as a cause of genital herpes. Abstinence-only sexual health classes, commonly taught in Virginia, generate young adults who are under-educated in sexual health, increasing STI risks. College students in southwest Virginia were surveyed to assess comprehensiveness of high school health education regarding HSV-1 and HSV-2 and to identify students’ preferred methods for STI education.

**Methods:**

To obtain data on knowledge of HSV, comprehensiveness of sexual health education in high school, and preferred learning methods, 237 college students participated in an online questionnaire and 28 students were interviewed using structured interviews.

**Results:**

Questionnaire and interview data indicated that Family Life Education classes need to include more comprehensive information on prevention, viral transmission, and differences between HSV-1 and HSV-2. The majority of total respondents (both the questionnaire and interview) (65%) reported non-comprehensive high school sexual health education. The majority of interview (79%) and questionnaire (55%) respondents wished they had learned more about herpes and other STIs in high school. Education preferences of both interviewed and surveyed respondents included interactive internet programs or games, more realistic lectures, and learning about STIs later in high school when students reported greater sexual activity.

**Conclusion:**

Our results indicate that more comprehensive sexual health education is needed and wanted by students in southwest Virginia. More relevant educational programs should be implemented for VA high school students utilizing technology and interactive methods to improve student engagement in sexual health education.

**Implications and contribution:**

These studies provide information on knowledge of herpes simplex viruses among college students, comprehensiveness of sexual health education received in high schools, and preferred methods to learn about HSV and other STIs. These studies inform the facilitation of improved health education practices and programs for high school and college students.

## Introduction

Sexually transmitted infections (STIs) are a serious and growing problem in the United States, particularly among adolescents and young adults. Nearly half of all newly acquired STIs are among young people aged 15–24, and approximately one in four sexually active teens contracts an STI every year [1]. Genital herpes, one of the most common STIs worldwide, is typically caused by herpes simplex virus 2 (HSV-2) infection, although herpes simplex virus 1 (HSV-1) may also cause genital lesions and is becoming more common in genital herpes.

Genital herpes is a non-reportable infection, with no widely adopted screening recommendations across geographical regions in the U.S. Prevalence data for HSV-1 and HSV-2 are thus unavailable for specific geographical regions in the U.S, particularly in rural areas, or for specific groups of people such as college students. The Centers for Disease Control (CDC) estimates 22% of adults in the U.S. are currently seropositive and approximately 776,000 new HSV-2 infections occur each year, 45% of which are in people ages 15–24[2] [3]. The National Health and Nutrition Examination Survey (NHANES) of 1999–2004 reported a prevalence increase from 1.6% among adolescents (14–19 years of age) to 10.6% among young adults (20–29 years of age), increasing to 22.1% in adults 30–39 [4, 5] [4]. [6]. This substantial increase in prevalence over a lifetime indicates that targeting adolescents and young adults for prevention efforts is the most effective way to lower the incidence of HSV-1 and HSV-2 in the U.S.

In Virginia, sexual education law and policy dictates that middle and high schools must administer Family Life Education, including a component of sex education that is abstinence-based [7]. Each school district has the option to include or exclude material related to sexual health, contraceptives, and STIs, as long as the school includes the importance of abstinence and delaying sexual activity. In addition, parents are given the opportunity to opt out children from any portion of the Family Life Education program [7]. Virginia’s flexible Family Life Education policy leaves large inconsistencies in sex education throughout the state, particularly in rural areas where the percentage of abstinence-only programs is frequently higher than in other regions [8].

Compared to older adults, sexually active adolescents and young adults are at a much higher risk of acquiring and spreading STIs [9]. This is often due to behavioral and biological differences in this age group, limited resources, and a general lack of knowledge about sexual health [9]. Existing evidence suggests that there is a general lack of knowledge on various aspects of HSV [10]. General knowledge of STIs, HSV included, are of particular concern in rural or semi-rural environments where poverty is present, there is increased stigma associated with STIs, comprehensive sexual health education is lacking, and access to health care is limited [11]. A previous study showed that only 40% of men whose current female partner had genital herpes felt well-informed about genital herpes [12]. Additionally, female college students felt more vulnerable to infection, viewed acquisition of the virus as a more negative event, and were more likely to think that their parents and their peers would think poorly of them compared to men in the same study [13]. Thus, educational programming needs to foster a better understanding of the virus and focus on lowering the stigma associated with the virus, particularly in rural populations.

The purpose of this study was to gain an understanding of the level of knowledge about HSV-1 and HSV-2 among college students in southwest Virginia. The study was also designed to identify how young people learn about STIs, including HSV, and to obtain their opinions on better ways of teaching adolescents and young adults about STIs. We identified gaps in overall knowledge of HSV, including a lack of understanding of the differences between HSV-1 and HSV-2 and incomplete knowledge regarding viral transmission. College students that participated in the study expressed a desire to learn more about STIs in high school and suggested a variety of options for improving sexual health education for adolescents and young adults.

## Materials and methods

### Demographics

Baseline demographic characteristics of the questionnaire study population were recorded. This includes sex, grade level or class, race/ethnicity, and number of total past sexual partners (Table 1).

**Table 1:** Demographic characteristics of questionnaire study participants. (n = 235).

Demographic Characteristics	Total Participants		Male		Female		Prefer not to respond (M/F)	
	n	%	n	%	n	%	n	%
**Total Participants**	235	100.0%	89	37.9%	143	60.9%	3	1.3%
**Grade Level or Class**								
High School Sophomore	1	0.4%	0	0.0%	1	0.4%	0	0.0%
College Freshman	17	7.2%	8	3.4%	9	3.8%	0	0.0%
College Sophomore	32	13.6%	14	6.0%	18	7.7%	0	0.0%
College Junior	34	14.5%	13	5.5%	20	8.5%	1	0.4%
College Senior	126	53.6%	45	19.1%	80	34.0%	1	0.4%
Graduate Student	24	10.2%	8	3.4%	15	6.4%	1	0.4%
No response (Grade/Class)	1	0.4%	1	0.4%	0	0.0%	0	0.0%
Totals	235		89		143		3	
**Race/Ethnicity**								
African American/Black	5	2.1%	2	0.9%	3	1.3%	0	0.0%
Asian	33	14.0%	14	6.0%	19	8.1%	0	0.0%
Hispanic/Latino	9	3.8%	3	1.3%	6	2.6%	0	0.0%
Multiracial	2	0.9%	0	0.0%	1	0.4%	1	0.4%
Native American/American Indian	2	0.9%	2	0.9%	0	0.0%	0	0.0%
Pacific Islander	1	0.4%	0	0.0%	1	0.4%	0	0.0%
White/Caucasian	178	75.7%	65	27.7%	112	47.7%	1	0.4%
Other	2	0.9%	2	0.9%	0	0.0%	0	0.0%
Prefer not to respond (Race/Ethnicity)	3	1.3%	1	0.4%	1	0.4%	1	0.4%
Totals	235		89		143		3	
**Number of Total Past Sexual Partners**								
Ten or more	24	10.2%	11	4.7%	12	5.1%	1	0.4%
Three to Nine	78	33.2%	26	11.1%	52	22.1%	0	0.0%
One to Two	82	34.9%	30	12.8%	52	22.1%	0	0.0%
Zero	41	17.4%	16	6.8%	25	10.6%	0	0.0%
Prefer not to respond (Sex Partners)	10	4.3%	6	2.6%	2	0.9%	2	0.9%
Totals	235		89		143		3	

### Questionnaire

College students (237) were recruited from Virginia Polytechnic Institute and State University (Virginia Tech) and Radford University. Virginia Tech’s undergraduate student body consists of 24,247 students and 17,655 of those (72.8%) are Virginia residents. Radford’s undergraduate student body consists of 8,885, and 8,417 of those students (94.7%) are Virginia residents. Participants were recruited through the use of flyers, class listservs, and VT Daily News announcements, for participation in an anonymous online questionnaire generated through Qualtrics (www.qualtrics.com). The questionnaire was distributed openly from February 01 –April 11^th^, 2014, and no subsequent response rate was determined. Institutional Review Board (IRB) information was provided on the first page of the online questionnaire and informed consent was implied by voluntary submission of the online questionnaire. The questionnaire consisted of 14 multiple choice questions pertaining to knowledge of HSV infection (1 question consisting of 8 sub-questions), past education on HSV and sexually transmitted infections, preferred methods for STI education, and demographics. The questionnaire was modeled after a similar study (13) performed in 1999 and modified to fit additional research questions and to best reflect modern understanding of these topics. Data analyses were performed using Qualtrics intrinsic analysis tools. Questionnaire questions can be found in Supplementary S1 File Questionnaire Questions.

### Interview

To obtain qualitative data to better understand online questionnaire responses, 28 participants were interviewed, including 6–8 from each class (college freshmen, sophomores, juniors, seniors). Interview participants were randomly selected during a concurrent study collecting and analyzing blood for HSV-1 and HSV-2 seroprevalence. The implications of the concurrent nature of this data collection suggest that participants may have been interested in the topic and/or concerned about their HSV status.

Interviews took place over a three-month period of time, spanning from February to April 2014. Participants were asked if they were willing to participate in the interview for a small compensation. Informed consent was obtained in writing from all interviewed participants. Interviewed participants were provided with the survey link to the online questionnaire and asked to complete it at a later time. The specific questionnaire responses of interviewed participants were not compared. Participants were asked 16 questions based on the questionnaire but with open-ended answers instead of multiple choice. Of these 16 questions, 11 were selected for inclusion in this report based on the significance and relevance of the findings. For example, one question asked the participant how upset they would be if they were to receive an HSV diagnosis, which was not relevant to the educational assessment.

Participant answers during interviews were recorded manually on a computer by the interviewer. Results from interviews were analyzed by identification of themes within the qualitative data. Themes in the data were identified through manual coding where the identification of passages of text or ideas could be labeled as examples of a thematic idea. This coding process allowed for the creation of a manually created matrix of thematic ideas that were examined together. These themes were used to support trends identified within the quantitative questionnaire data. Interview data can be found in Supplementary S2 File Interview Data Q1-11.

### Institutional review board

All studies were approved by the review boards of Virginia Tech (IRB #14–092 and 13–1058) and Radford University (IRB# 571731–1).

### Limitations of research methodologies

Limitations of the questionnaire portion of the research study include the inability to calculate a response rate. The participants represent only a small portion of students that may have attended Virginia high schools, thus these results cannot conclude the level of education currently present. Furthermore, participants were not asked if they attended a Virginia high school, and it is assumed that the study population follows a similar status as the in-state population of both schools (Virginia Tech 72.8% and Radford University 94.7%).

The questionnaire was piloted with this population but a reliability coefficient was not calculated due to limited access to participants.

Limitations of the interview portion include the inability to record participants’ responses due to the sensitive nature of the discussion. Researchers manually typed the responses into a computer, which may have limited the conversational flow of the interview. Due to resources, the number of interview participants was limited. Additionally, there is an element of participation bias in selection for interviewees, as participants were randomly selected from a group participating in the associated HSV seroprevalence study.

## Results

### General knowledge of HSV

**Fig 1:**
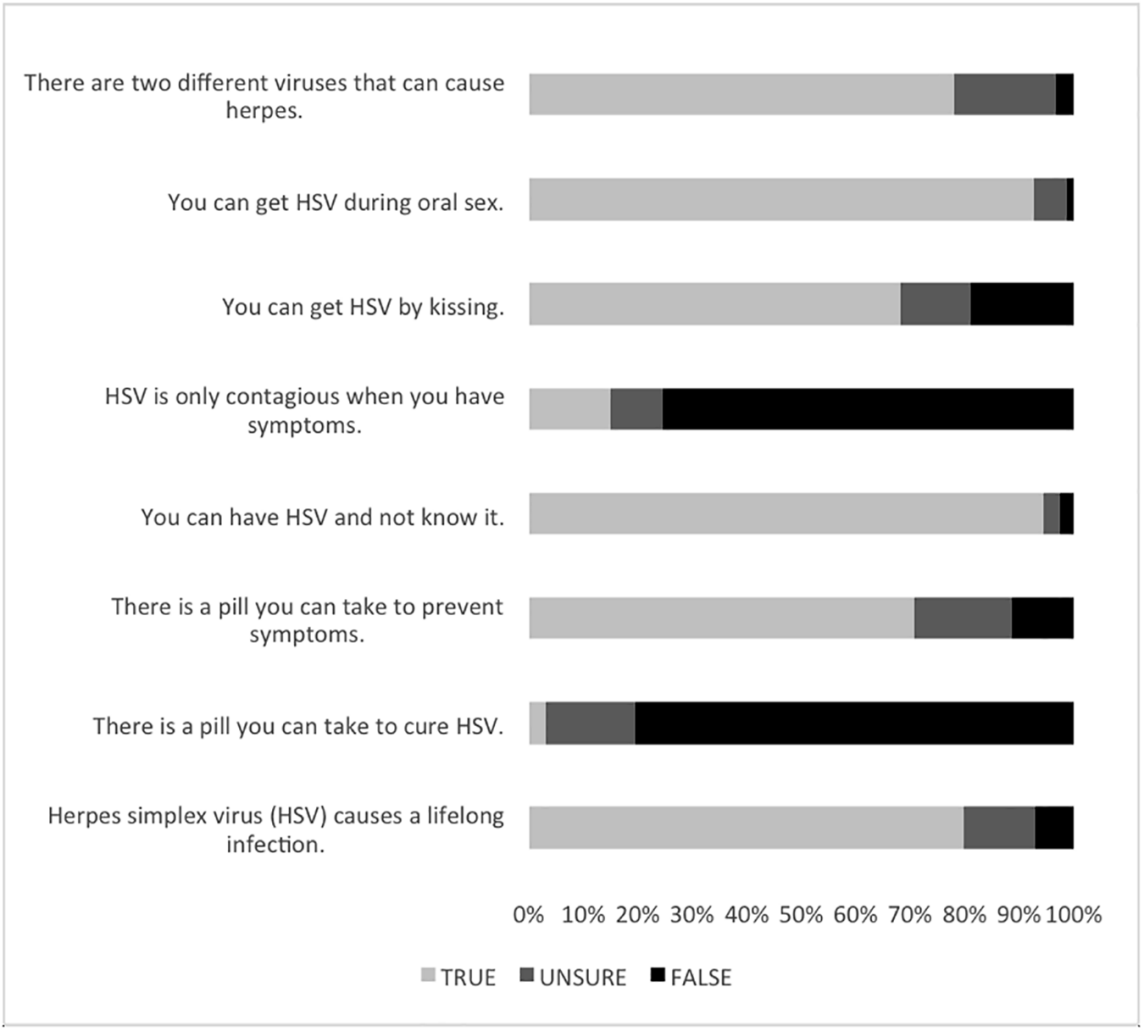
General knowledge of HSV. Approximately 70–95% of respondents answered the knowledge-based question about HSV correctly, indicating that respondents’ knowledge of HSV was relatively comprehensive but may include some gaps in understanding. Data represent responses to a single knowledge-based question, consisting of 8 sub-questions. (n = 235–237).

The questionnaire respondents’ general knowledge of HSV was relatively comprehensive (Fig 1). Approximately 80% (79.8%, n = 237) of respondents knew that herpes simplex causes a lifelong infection. The majority of respondents (80.4%, n = 235) correctly answered False when asked if there is a pill you can take to cure HSV, and 70.6% (n = 235) of respondents were aware that there is a pill you can take to prevent symptoms. Most (75.4%, n = 236) respondents were aware of asymptomatic contagiousness and 92.8% (n = 235) were aware that HSV can be acquired during oral sex. Nearly all respondents were aware that a person can have HSV and not know it (95.3% of 237 respondents) and that a person can become infected with HSV during oral sex (93.1%).

In sub-questions regarding knowledge, there was no substantial difference in female and male responses. Nearly 20% (18.6%, n = 236) of respondents were not sure there were two different viruses that cause herpes. Males tended to be slightly less certain than females about how a person can become infected with HSV, answering “Not Sure” more frequently on sub-questions that address contagiousness when symptoms are present and becoming infected with HSV during kissing or oral sex. Additionally, 60% of interviewed participants did not know or were unsure of the difference between the two viruses. Only 68.5% of respondents thought that a person could become infected with HSV by kissing, which may indicate that many college students do not realize that cold sores are caused by HSV-1 or that they are simply unaware that HSV-1 can be shed orally.

### Sexual health education

Respondents were asked to self-report their level of sexual health education. Two thirds of all participants (65.1%) had limited or no education on STIs in middle and high school, indicating that the majority received non-comprehensive sexual health education. Additionally, 24% of respondents reported that genital herpes was not discussed at all in their sexual health education classes. Only 14% of interviewed participants indicated that they had received comprehensive information on HSV in their sexual health education classes, and half of participants could only name three or fewer sexually transmitted infections they had learned about in high school. More than half of questionnaire respondents indicated that they wished they had learned more in middle and high school. Similarly, interviewed participants overwhelmingly (79%) responded positively when asked if they wished that they had learned more.

**Fig 2:**
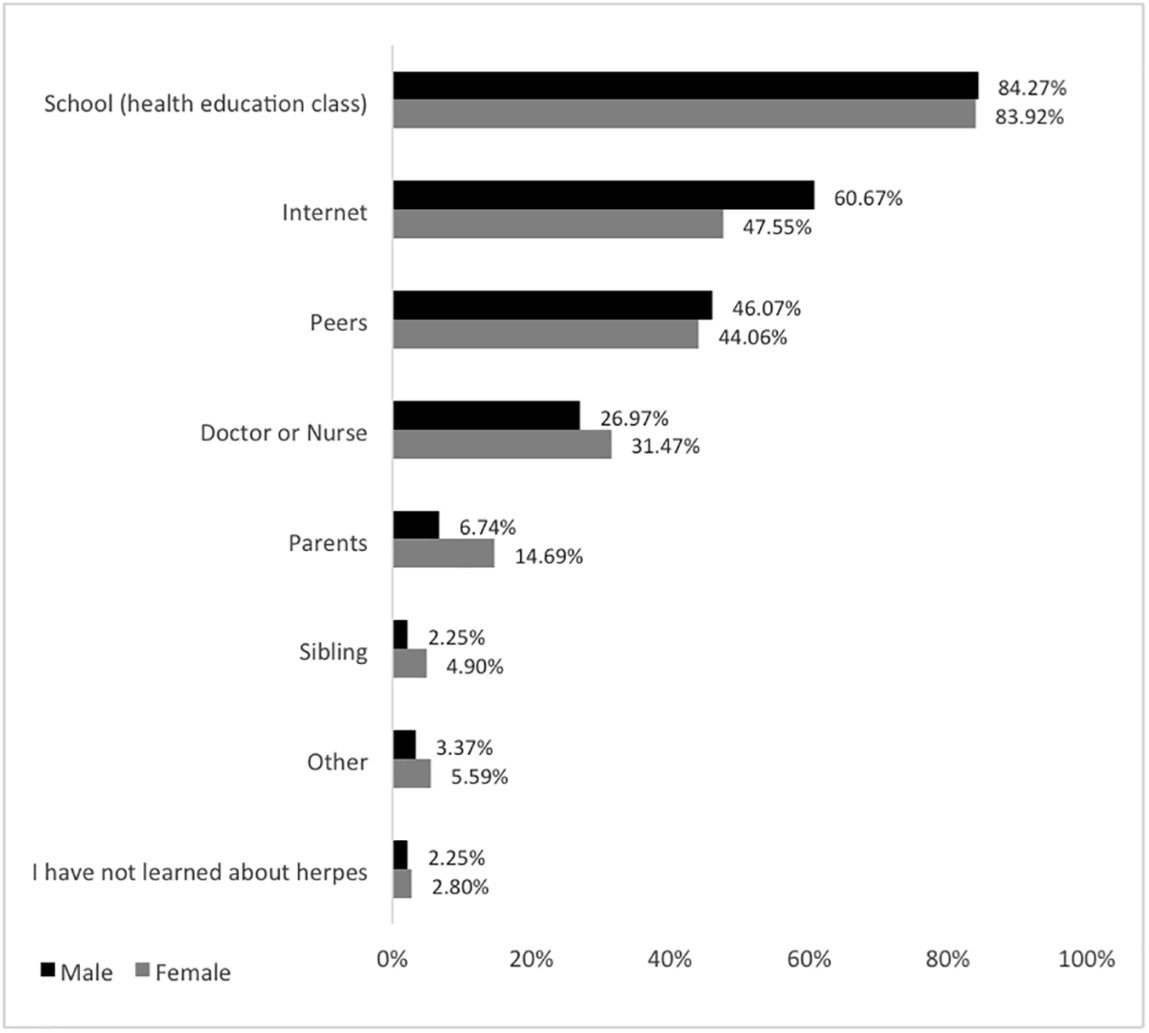
Resources used by respondents to learn about herpes. The majority of respondents have learned about herpes from health education classes in school, from the internet or from peers. (n = 237).

To gain an understanding of how adolescents and young adults obtain information about HSV and other STIs, participants were asked where they have learned about HSV and the resources they use to find information, with the option of selecting more than one source (Fig 2). The majority (84%) of both females and males obtained information from school sexual health education classes, indicating that this is the primary resource that young people use in information acquisition regarding STIs. Additionally, 61% of males and 48% of females used the internet to learn about genital herpes, demonstrating that the internet is approaching school health education classes as a primary source for STI education among young adults. Approximately 45% of respondents obtained information about genital herpes from their peers, with no notable difference in gender. Only about a third of respondents (30%) indicated that medical personnel were their source if information and only a few indicated parents or siblings.

**Fig 3:**
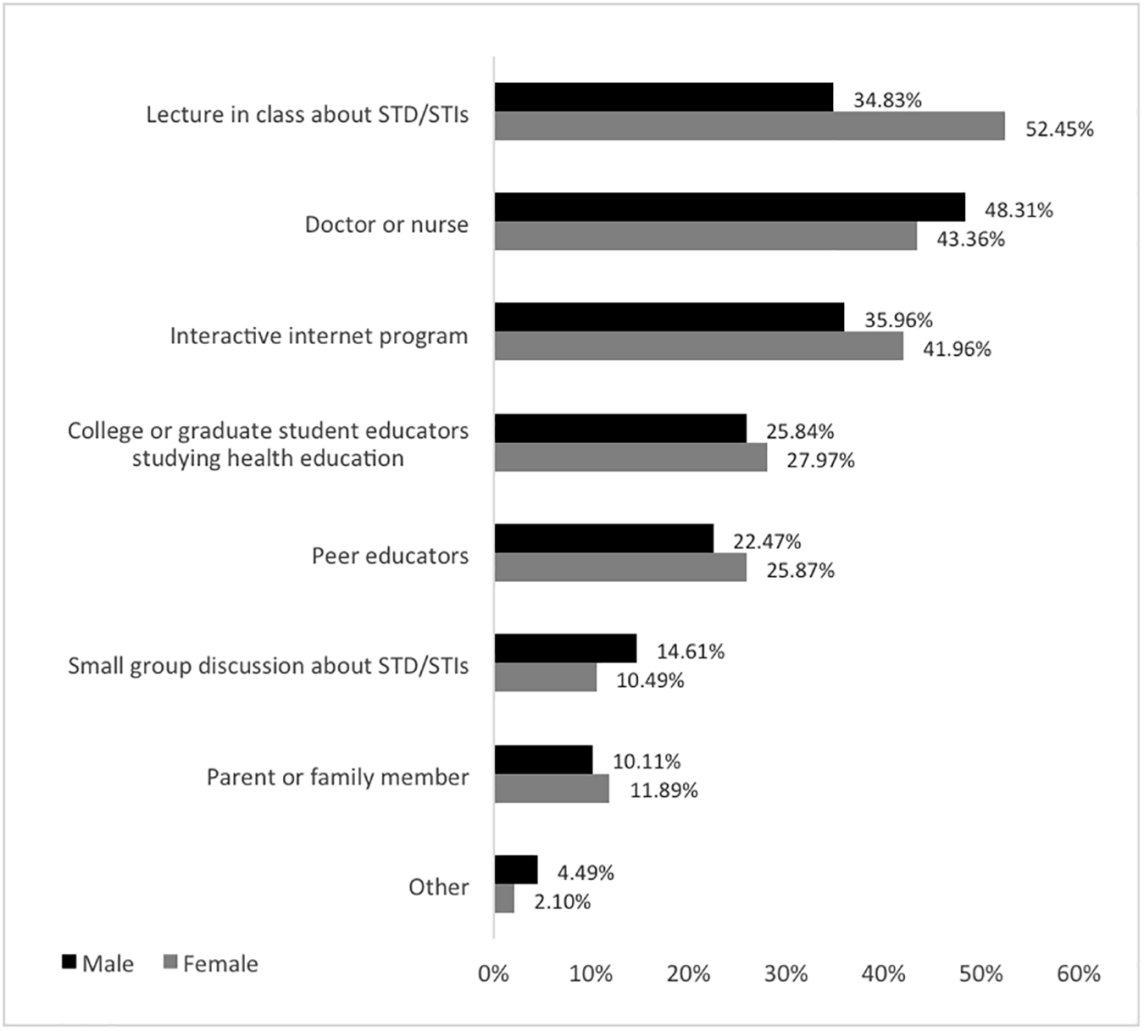
Preferred methods for questionnaire respondents to learn about STIs and sexual health. The majority of respondents indicated a preference to learn either from a doctor or nurse, an interactive internet program, or from a lecture in class about STD/STIs. (n = 237).

**Fig 4:**
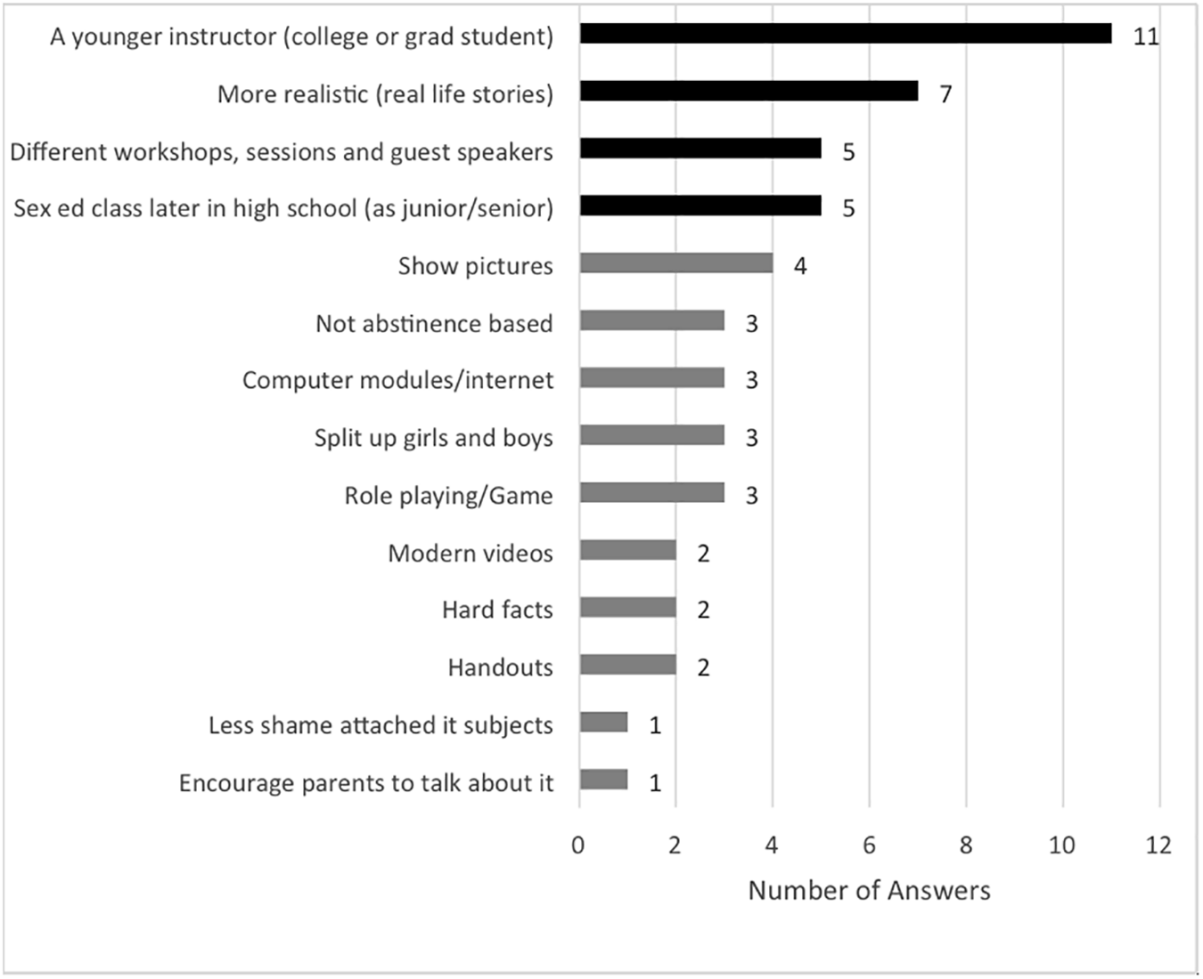
Interview respondents’ ideas for how to change the way sexual health education is taught in schools. Interview participants indicated a preference to learn from a younger instructor, have realistic lectures with real life stories, have workshops and guest speakers during sex education class, and to have sexual health education taught in the later years of high school (junior or senior year) because that was when they were more sexually active. (n = 26).

Participants were asked to identify ways to better teach students about STIs and sexual health in high school based on their own preferences (Fig 3). Questionnaire respondents’ preferences for methods of learning about genital herpes and other STIs indicated that the majority of people prefer to learn from a doctor or nurse, although this response did not correlate with the previous question, in which only a third of respondents indicated that they relied upon medical personnel for information regarding STIs. Other preferred methods were through an interactive web-based program, or from a lecture in class about STIs, although females showed a greater preference for in-class lectures than males. Preferences expressed by interviewed participants supported and expanded the data, responding that they would prefer a younger instructor, such as a college or graduate student, for in-class lectures in high school. Additionally, participants suggested more realistic lectures with real life stories, workshops, and guest speakers during sex education class (Fig 4). Interviewees also indicated that having sexual health education would be more beneficial if it was taught in the later years of high school (junior or senior year) because that was when they were more sexually active. Taken together, these results indicate a desire for more relevant sexual health education.

### Self-reported prevalence

**Fig 5:**
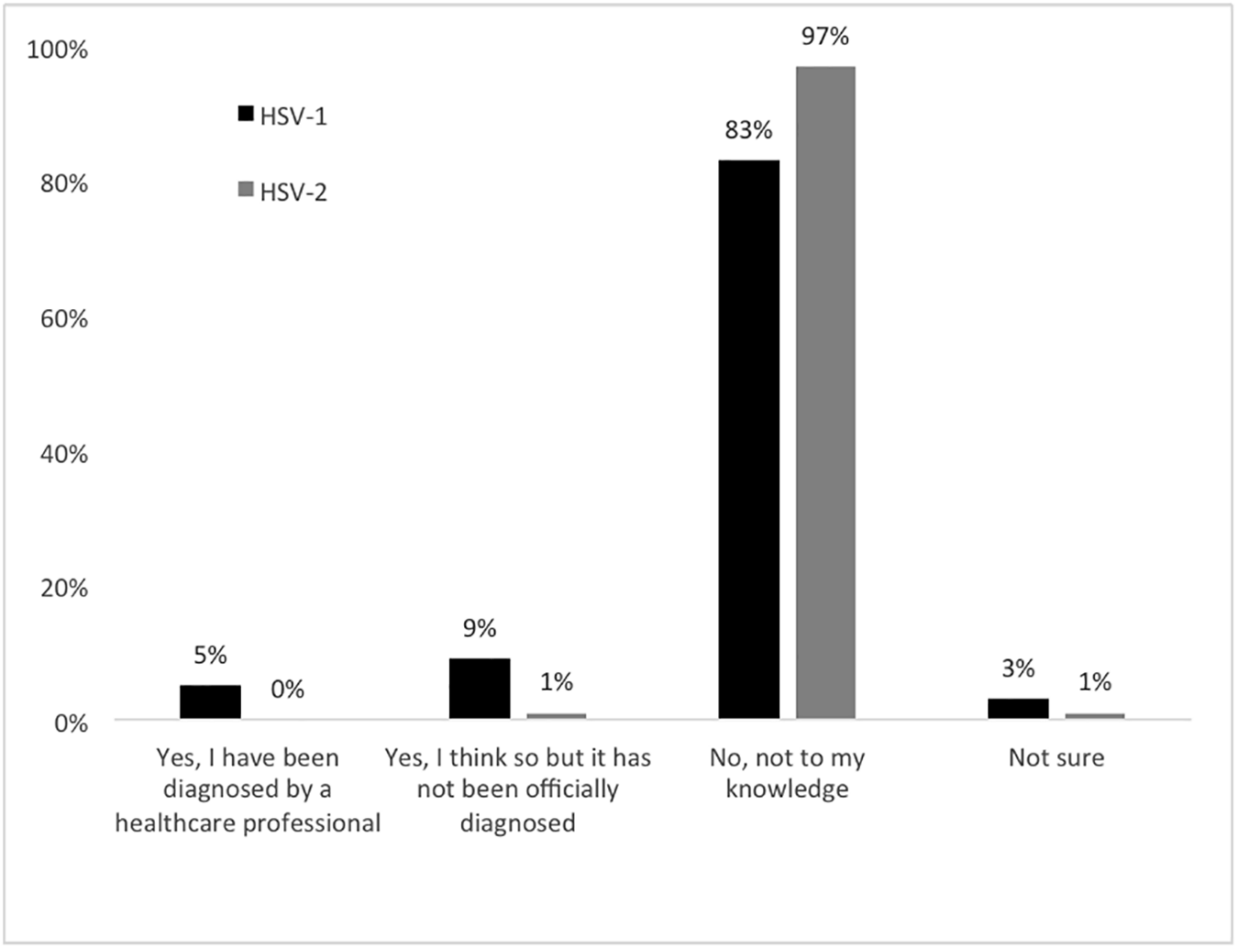
Self reported infection of HSV-1 and HSV-2. The majority of respondents self-reported that they did not have either HSV1 or HSV2. (n = 236).

Respondents were asked to self-report whether or not they had HSV-1 and/or HSV-2. Fourteen percent (14%) of respondents self-reported as HSV-1 positive, either by diagnosis from a healthcare professional or believing they had it without an official diagnosis (Fig 5). Only 1% of questionnaire respondents reported as HSV-2 positive. The most recent available national prevalence rates (2010) show that in this age group, between 49% and 52% of the population are HSV-1 seropositive and 12% to 15% are HSV-2 seropositive, with up to 90% of infected individuals unaware that they have the viruses [14, 15]. Thus, our data indicate that a large number of college students may be unaware of their HSV-1 and HSV-2 status, which can contribute to viral transmission among young adults.

## Discussion

Due to current sexual health education policies in Virginia, many adolescents receive inadequate sexual health education in middle and high school. These issues necessitate strategies to identify the burden of disease in Virginia, as well as identify better ways to teach about sexually transmitted infections. This study was an attempt to understand the level of knowledge of HSV-1 and HSV-2 among young adults and the impact of sexual health education policies on these infections in rural southwest Virginia.

Our data on general knowledge of HSV stress the importance of necessary changes in the current sexual health education curriculum. The majority of participants received a non-comprehensive sexual health education in high school, with many indicating that they do not feel well-informed on STIs. Knowledge level of HSV among participants was encouraging in regards to permanence of infection, ability to medicate for symptoms, and contagiousness of asymptomatic individuals. Respondents were also aware that it is possible to have HSV and not know it, which is supported by the self-reported prevalence data. However, gaps in participant knowledge of HSV were evident, particularly in differences between HSV-1 and HSV-2 and transmission routes. A study on HSV-2 knowledge in urban college students found similar results regarding college students’ knowledge level related to asymptomatic infections and contagiousness [13]. Thus, college students in both rural and urban areas may be aware of several important aspects of the diseases that can help to reduce transmission, but are still lacking in some details about HSV infections. Participants in our study overwhelmingly indicated that they wished they had learned more about sexual health, HSV, and other STIs, suggesting that adolescents and young adults would be receptive to an increase in sexual health education in school using updated teaching techniques. However, further studies are needed to expand upon both studies to better understand current differences between urban and rural youths and young adults regarding these topics.

A large percentage (83%) of questionnaire respondents stated that they received their information on STIs from their sex education class in high school, pointing to the importance of a comprehensive school-based sexual health curriculum. Many respondents, especially females, noted a preference to learn in a classroom setting. Students have a multitude of ideas about how sexual health education can be improved and overall interview results indicate that students feel disconnected from the current curriculum. During interviews, suggestions for ways to improve sexual health education included preferences for relatable instructors that utilize realistic examples, interesting workshops, guest speakers, role-playing, games, and splitting up females and males so open conversation can take place. Additional preferences include continued education during junior and senior years, when students are more sexually active. Sexual health or family life education programs can be re-designed to ensure that the teaching style and curriculum is more relevant for engaging and informing high school and college students.

Students also indicated that they have learned about HSV and STIs from their peers, addressing an important preference to learn about sensitive and stigmatized subjects such as STIs from someone closer to their own age to whom they can relate. Two of the identified preferred learning methods, from a doctor or website resources, suggest that many people want to learn about STIs on an individual basis in a confidential manner. This finding is expected due to the significant stigma associated with STIs and hesitance to discuss genital herpes and other STIs [10, 16]. However, members of this age group often feel uncomfortable acknowledging infections acquired through sexual activity and often do not seek necessary health care for fear of confidentiality. The social stigma associated with STIs often prevents teens and young adults from discussing STIs and sexual health with healthcare workers and sexual partners, leading to continued spread of infections. Providing preventative health education about STIs using modern, culturally appropriate, and relevant pedagogical methods, designed to break through social stigma, is essential to reduce the prevalence of genital herpes and other STIs in adolescents and young adults.

Along with other researchers [13], we found that many students learn about sexual health and STIs through internet resources. To address the need for accurate web-based information, and current learning preferences of this age group, self-directed internet-based educational modules may be a solution. The wide range of communication platforms presented by available technology may serve as useful tools for engaging adolescents in sexual health education. Studies have shown positive outcomes in using online resources and computer-based forms of sexual health education, including significant decreases in adolescent sexual activity, increased condom use, and knowledge of essential concepts [17–19]. Additionally, these programs can provide the opportunity to target educational interventions for specific geographic locations and at-risk populations [20]. Research on the incorporation of this educational method into public school curricula provides encouraging outcomes to support wide-range application, including in Southwest Virginia [21, 22]. In addition, the implementation of web-based programs shows promising cost-benefit effectiveness for school systems, where measurable benefits in STI reductions highly justify the low cost of this format [21].

Similar to existing web-based programs, the development of an interactive online sexual health education course as a part of the school curriculum may provide significant opportunities for improved learning and reduced barriers. Such a course may include multiple lectures and interactive online labs, developed and recorded by relatable, energetic doctors or public health professionals. The online program may be formatted into physical education curricula each year of high school, providing information relevant to each grade level based on students’ changing levels of personal and sexual maturity.

In conclusion, this study has identified gaps in the general knowledge of HSV infections, a lack of comprehensiveness in sexual health education, and students’ preferred methods for learning about genital herpes and other STIs. We conclude that innovative methods need to be used more widely in school curricula, to provide accurate and relevant sexual health information in a format that addresses the ways students prefer to learn about these sensitive topics. Our study indicates that students are eager to learn more about sexual health and sexually transmitted infections, which is a valuable finding when considering integration and acceptance of new methods for STI education. Through the use of technology and comprehensive educational programs that will engage students, it is possible to improve access to quality health information and STI prevention services to young people in rural Virginia. The development of interactive courses for adolescents and young adults that incorporates the use of younger instructors, realistic scenarios, open discussion forums, and continuous education throughout high school would provide students the information they need, when they need it the most, and a well-rounded view of sexual health.

## Supporting information

S1 File Questionnaire questions. File contains questions asked of participants in the online questionnaire.
(PDF)

S2 File Interview data Q1-11. File contains questions and answers asked of interview participants. The number and percentage of each response are included.
(PDF)
