# Supplementary material for: Herpes simplex virus 1 and 2 educational assessment of young adults in rural southwest Virginia
Source: PLoS One. 2017 Jun 27;12(6):e0179969. doi: 10.1371/journal.pone.0179969 (PMC5487059; doi:10.1371/journal.pone.0179969)
Supplement: S1 File — File contains questions asked of participants in the online questionnaire. (PDF) [file pone.0179969.s001.pdf]

Questions	Answer Options
1a. Herpes simplex virus (HSV) causes a lifelong infection.	-True<br>-False<br>-Unsure
1b. There is a pill you can take to cure HSV.	
1c. There is a pill you can take to prevent symptoms.	
1d. You can have HSV and not know it.	
1e. HSV is only contagious when you have symptoms.	
1f. You can get HSV by kissing.	
1g. You can get HSV during oral sex.	
1.f There are two different viruses that can cause herpes.	
2. How would you describe the level of sexual health education you received in middle/ high school?	- Very comprehensive (education included contraceptive use, sexually transmitted infections (STIs), healthy relationships, human reproduction etc.)<br>-Somewhat comprehensive (education included some contraceptive use, and information on reproduction but limited STIs)<br>-Not comprehensive (abstinence-only programs taught)
3. Was Herpes Simplex Virus, specifically genital herpes, discussed in your sexual health education class?	-Yes<br>-No<br>-Prefer not to respond
4. Was Herpes Simplex Virus, specifically genital herpes, discussed among your peers in high school?	-Yes<br>-No<br>-Prefer not to respond
5. Where have you learned about herpes? (Check all that apply)	School (health education class)<br>-Doctor or nurse<br>-Peers<br>-Parents<br>-Sibling<br>-Internet<br>-I have not learned about herpes
6. Do you wish that you had learned more about herpes (and other STIs) in your middle and high school sexual health education classes?	-Yes<br>-No<br>-Prefer not to respond
7. What would be the easiest way for you to learn about STIs and sexual health?	-Peer Educators<br>-College or graduate student educators studying health education<br>-Doctor or nurse<br>-Parent or family member<br>-Interactive Internet program<br>-Lecture in class about STIs<br>-Small group discussion about STIs<br>-Other ___________________
8. If you found out you had genital herpes, who would you talk to to learn more about it?	-Friends<br>-Sexual partner<br>-Doctor or nurse<br>-Parent<br>-Sibling<br>-School counselor or health educator<br>-Other ____________________

9. Do you have HSV1 (commonly characterized by fever blisters/cold sores)?	-Yes, I have been diagnosed by a healthcare professional<br>-Yes, I think so but it has not been officially diagnosed<br>-No, not to my knowledge<br>-Not sure
10. Do you have HSV2 (commonly characterized by genital sores)?	-Yes, I have been diagnosed by a healthcare professional<br>-Yes, I think so but it has not been officially diagnosed<br>-No, not to my knowledge<br>-Not sure
11. Gender:	-Female<br>-Male<br>-Transgender<br>-Prefer not to respond
12. Race/Ethnicity:	-African American/Black<br>-Asian/Pacific Islander<br>-Hispanic/Latino<br>-Multiracial<br>-Native American/American Indian<br>-White<br>-Other____________<br>-Prefer not to respond
13. Class status:	-College Freshman<br>-College Sophomore<br>-College Junior<br>-College Senior<br>-Graduate / professional student<br>-Other ____________________
14. Number of total past sexual partners:	->10<br>-3-9<br>-1-2<br>-0
