# Supplementary material for: Herpes simplex virus 1 and 2 educational assessment of young adults in rural southwest Virginia
Source: PLoS One. 2017 Jun 27;12(6):e0179969. doi: 10.1371/journal.pone.0179969 (PMC5487059; doi:10.1371/journal.pone.0179969)
Supplement: S2 File — File contains questions and answers asked of interview participants. The number and percentage of each response are included. (PDF) [file pone.0179969.s002.pdf]

Interview Data: 28 total participants

Q1	Question 1) Do you know what disease HSV1 and HSV2 cause?		
	Yes	9	32%
	No	10	35%
	Answered Partially correct	2	7%
	Unsure in difference between HSV1 and HSV2	7	25%
Q2	Question 2) How do you catch it?		
	Answered correctly	12	43%
	Answered incorrectly	1	3%
	Unsure	3	11%
	Partially correct (only mentioned sex)	12	43%
Q3	Question 3) How do you get rid of HSV?		
	Answered correctly	21	75%
	Answered incorrectly	4	14%
	Unsure	3	11%
Q4	Question 4 Did you learn about HSV in your high school health ed class? And what did you learn?		
	Yes, comprehensive	4	
	Yes, partial	16	
	No	3	
	Unsure	5	
Q5	Question 5) Did you learn about other STIs, and which ones?		
	Yes, Comprehensive (more than 4)	10	
	Yes, partial (3 or less)	13	
	No	1	
	Unsure or Doesn't remember	4	
Q6	Question 7) Do you wish you had learned more about HSV and Stis in sex ed in high school?		
	Yes	22	
	No	6	
Q7	Question 8) Was HSV discussed with peers?		
	Yes	8	
	Yes, jokingly	7	
	No	13	
Q8	Question 9) Where have you learned about HSV and other STIs?		
	Peers	6	
	Internet (WebMD, VDH)	17	
	Nurse/doctor	2	

	Sex ed class	3
	TV	3
	College classes	9
	Schiffert health center	2
	Parents	2
	Books	1
Q9	Question 10) Best way to teach about STIs and sexual health in high school	
	Role playing or game	3
	handouts	2
	younger instructor (college or grad students)	11
	Split up girls and boys	3
	Sex ed class taught later in high school	5
	More realistic (real life stories)	7
	enourage parents to talk about it	1
	Different workshops, sessions and guest speakers	5
	Hard facts	2
	Show pictures	4
	Modern videos	2
	Computer modules/internet	3
	less shame attached it subjects	1
	not abstinence based	3
Q10	Question 12) Who would you go to for support and to learn more about it?	
	Doctor	15
	Friends	11
	Family Member	11
	Internet	9
	Partner	3
	Unsure	2
Q11	Question 13) Do you have HSV1 or HSV2?	
	HSV1	5
	HSV2	0
	No	21
	Unsure	4
